# Genetic and Epigenetic Alterations in Primary–Progressive Paired Oligodendroglial Tumors

**DOI:** 10.1371/journal.pone.0067139

**Published:** 2013-06-24

**Authors:** Lu-Ting Kuo, Shao-Yu Tsai, Cheng-Chi Chang, Kuang-Ting Kuo, Abel Po-Hao Huang, Jui-Chang Tsai, Ham-Min Tseng, Meng-Fai Kuo, Yong-Kwang Tu

**Affiliations:** 1 Division of Neurosurgery, Department of Surgery, National Taiwan University Hospital, Yun-Lin branch, Yun-Lin County, Taiwan; 2 Department of Nursing, College of Medicine, National Taiwan University, Taipei, Taiwan; 3 Graduate Institute of Oral Biology, School of Dentistry, National Taiwan University, Taipei, Taiwan; 4 Department of Pathology, National Taiwan University Hospital, Taipei, Taiwan; 5 Division of Neurosurgery, Department of Surgery, National Taiwan University Hospital, Taipei, Taiwan; University of Navarra, Spain

## Abstract

The aim of the present study was to identify genetic and epigenetic alterations involved in the progression of oligodendroglial tumors. We characterized 21 paired, World Health Organization (WHO) grade II and III oligodendroglial tumors from patients who received craniotomies for the partial or complete resection of primary and secondary oligodendroglial tumors. Tumor DNA was analyzed for alterations in selected genetic loci (1p36, 9p22, 10q23–24, 17p13, 19q13, 22q12), isocitrate dehydrogenase 1 (IDH1), isocitrate dehydrogenase 2 (IDH2) and the CpG island methylation status of critical tumor-related genes (MGMT, P16, DAPK, PTEN, RASSF1A, Rb1). Alterations of these markers were common early in the tumorigenesis. In the primary tumors we identified 12 patients (57.1%) with 1p36 deletions, 17 (81.0%) with 19q13 deletions, 9 (42.9%) with 1p36/19q13 codeletions, 11 (52.3%) with 9p22 deletions, and 12 (57.1%) with IDH1 mutation. Epigenetic analysis detected promoter methylation of the MGMT, P16, DAPK, PTEN, RASSF1A, and Rb1 genes in 38.1%, 19.0%, 38.1%, 33.3%, 66.7%, and 14.3% of primary tumors, respectively. After progression, additional losses of 1p, 9p, 10q, 17p, 19q and 22q were observed in 3 (14.3%), 1 (4.8%), 3 (14.3%), 2 (9.5%), 1 (4.8%) and 3 (14.3%) cases, respectively. Additional methylations of the MGMT, P16, DAPK, PTEN, RASSF1A, and RB1 promoters was observed in 4 (19.0%), 2 (9.5%), 0 (0%), 6 (28.6%), 2(9.5%) and 3 (14.3%) cases, respectively. The status of IDH1 mutation remained unchanged in all tumors after progression. The primary tumors of three patients with subsequent progression to high-grade astrocytomas, all had 9p deletion, intact 1p, intact 10q and unmethylated MGMT. Whether this may represent a molecular signature of patients at-risk for the development of aggressive astrocytomas needs further investigation.

## Introduction

Oligodendroglial tumors are slow growing tumors of the central nervous system that are common in the frontal and temporal lobes of the brain, which, include WHO grade II and III oligodendrogliomas and oligoastrocytomas. The survival of patients with oligodendroglial tumors varies greatly. Many of these neoplasms respond to chemotherapy, especially procarbazine-vincristine-CCNU (PCV) or temozolomide regimens. However, progression of oligodendroglial tumors to aggressive phenotypes or higher grade, e.g, astrocytomas, is also common and associated with much shorter survival times. Although histopathology remains the gold standard for oligodendroglioma diagnosis and grading, molecular diagnosis has become a useful method for neuropathologic evaluation of these tumors to predict the clinical outcome.

Previous research has identified genetic and epigenetic alterations involved in oligodendroglioma progression. For example, loss of 1p/19q is considered a common early event in the tumorigenesis of oligodendroglial tumors and has been linked to the prolonged survival times and chemosentivity of these patients [1]–[3]. Mutation of isocitrate dehydrogenases 1 and 2 genes (IDH1 and IDH2), which catalyze the oxidative decarboxylation of isocitrate to a-ketoglutarate, has been associated with improved survival in glioma patients [4], [5]. Also, O^6^-Methyl guanine methyl transferase (MGMT) a DNA repair enzyme, and methylation of the promoter of the *AGT* gene at 10q26 leads to the repression of gene expression, and enhanced chemosensitivity in gliomas, especially glioblastoma multiforme [6]–[8]. Our previous study, which analyzed 49 patients with oligodendroglial tumors, demonstrated that novel genetic and epigenetic alterations, other than 1p/19q loss, in the primary tumors also predicted progression-free or overall survival [9]. However, only a limited number of genetic loci have been investigated in regard to alterations between the primary and secondary tumors [2], [10]–[14], and no previous publication has evaluated the changes in the methylation status of tumor-related genes, other than MGMT, along the course of tumor progression [11].

The present study was a longitudinal investigation of 21 clinically, genetically and epigenetically-paired surgical specimens from resections of primary (at initial diagnosis) and secondary (after progression) oligodendroglial tumors. These paired samples were examined for changes in phenotype, chromosomal deletions, IDH1 mutation, IDH2 mutation and methylation status of tumor-related gene promoters.

## Materials and Methods

### Clinical Data

The study included 21 patients, all of Chinese origin, with 42 oligodendroglial tumors (oligodendroglioma grade II and III, oligoastrocytoma grade II and III) diagnosed according to the WHO criteria, after surgery, at the National Taiwan University Hospital (NTUH) between January 1996 and December 2005. All patients underwent two craniotomies for partial or subtotal tumor resection. Subtotal resection was defined when more than 95% of the tumor was removed, and partial resection was defined as less than 95%. The first tumors excised from patients were primary tumors; the second tumors were resected following tumor progression, defined as ≥25% increase in the largest cross-sectional area of the tumor. The histopathology of archival tumor specimens was reviewed by two pathologists who were unaware of patient data. Axial cross sections of magnetic resonance T1-weighted images with and without contrast were used to assess the tumor location and volume. Clinical data, including initial presentation, sex, age, extent of surgery, post-operative chemotherapy, irradiation, time to progression, and outcome, were obtained from the medical records. All participants provided their written consent to participate in this study, which was approved by the committee on human studies of NTUH (National Taiwan University Hospital).

### DNA Extraction and Quantitative Microsatellite Analysis for Genetic Diagnosis

DNA was extracted from paraffin-embedded tissues using a Genomic DNA mini Kit (Geneaid). The microsatellite markers used to identify genetic alterations included: D1S214, D1S463 and D1S507 on 1p; D9S162 and D9S285 on 9p; D10S185 and D10S192 on 10q; D17S786 and D17S1828 on 17p; D19S408 and D19S606 on 19q; and D22S421 on 22q. Quantitative microsatellite analysis (QuMA), using Taqman real-time polymerase chain reaction (PCR) with an annealing temperature of 59°C, is based on amplification of microsatellite loci that contain (CA)_n_ repeats, where the repeat itself is the target for hybridization by the fluorescence-labeled probe (CACACACACACACACACACACACACACAC). The primers for each microsatellite marker were designed using Primer Express (ABI). A detailed list of these primers is given in Table 1. A reference pool of six chromosome loci that remain unaltered during glioma tumorigenesis was used as controls. These included D2S385, D3S1554, D5S643, D8S1800, D12S1699 and D21S1904 [15]. The primers and probe were purchased from Purigo and ABI. The pooled standard deviation for all markers in normal DNA was used to calculate a tolerance interval (TI). In this study, copy numbers <1.45 were considered to be losses [9].

**Table 1:** Summary of primer sequences used for real-time PCR.

Microsatellite	Forward primer	Reverse primer
D1S214	GCCCGAATGACAAGGTGAGA	CATTCTGCATTCCTAAAAGCCAGTA
D1S463	GCCTGAAGCAATGAATAACAGTTG	CTTTTAAGCCTTTTAGTTAGTCTGAGTTTGT
D1S507	GAAAGCCACAAACCCTCTTCAC	GGATGGGCTCTAGGGTTTCTG
D9S162	CCAGAGAAACAGAACCAATAGAATACATAC	TCCCACAACAAATCTCCTCACA
D9S285	AAATTTGCCAAGAGAGTAGATCTGAAG	CCAATTAACATATCCCTCACCTTTG
D10S185	TTTCATTTGCCACTTTTCCTACATC	AAAATGGAAAAAGGGCAATGC
D10S192	GCTTACCTAGACCTTCATAATCACAGATG	TCAGACCAGTTTCCTGCCTTTATC
D17S786	GAGAGTGAAAGTGACATGTTTTCCA	ATTTGGGCTCTTTTGTAAAGAATTCT
D17S1828	ACAGGTGCACTCACAGATTTGC	AGACTCTTTTTGGAGATTCAGGGTAA
D19S408	CGCAAGCCTGAAGTATGTGCTA	GAGAACCAACTCATCTTTATTAAATGCA
D19S606	CCCTCCGTGGGCACTGT	AGGTACGAGGCTGTGCCTGTAG
D22S421	AAAACCTGCTGCCCCTAACAT	GCCTTTCTTAAAGGAATTTCATACAACT

### PCR Amplification and Direct Sequencing of IDH1 and IDH2

PCR amplification was performed to amplify a region of IDH1, including codon 132, and IDH-2, including codon 140 and 172 regions. Primers (forward-5′- GTGCTAAAACTTGGCAGATT and reverse-5′- CCTTGCTTAATGGGTGTAGA) were designed to amplify a 660-bp fragment spanning the catalytic domain of IDH1, and primers (forward-5′- TGCTTGGGGTTCAAATTCTG and reverse-5′- ACTGGATCTCCTCGCCTA) were designed to amplify a 294-bp fragment of IDH2. Reactions contained 4 µl (15–400 ng) gDNA template, 4.4 µl nuclease-free H2O, 0.8 µl forward and reverse primers, 10 µl 2X master mix buffer. The PCR program consisted of a 10 minute initial denaturation step (94°C), followed by 35 cycles of 94°C for 60 seconds, 58°C for 60 seconds and 72°C for 60 seconds. This was followed by a final extension step consisting of 10 minutes at 72°C. PCR products were sequenced directly by the Missionbio Company. The resulting sequences were analyzed with Softgenetics Mutation Surveyor DNA Variant Analysis Software.

### Promoter Methylation Status of Tumor-related Genes

The methylation status of six tumor-related genes was determined from tumor DNA using methylation-specific PCR (MSP). One microgram of genomic DNA from each sample was modified by treatment with bisulfite [16]. MSP was performed using specific primers for either methylated or unmethylated DNA, which was modified by bisulfite treatment. The PCR reactions were carried out using previously published primer sets and optimized conditions for MGMT, p16, Death-associated protein kinase (DAPK), phosphatase and tensin homologue deleted on chromosome ten (PTEN), RAS association domain family 1A (RASSF1A) and the retinoblastoma gene (Rb1) [7], [17]–[20]. PCR amplification was performed in a thermal cycler with a 15-minute denaturation at 95°C, followed by 40 cycles of 94°C for 45 seconds, 55–66°C for 45 seconds, and 72°C for 1 minute. This program was followed by a final extension at 72°C for 20 minutes. A negative control without a template, a generalized negative control, and a generalized positive control were used for each MSP set. The PCR products were separated on 4% agarose gels, stained with ethidium bromide and visualized under UV illumination. If the amplicon for both the M- and U-primer or for the M-primer alone could be identified, the promoter of a gene was considered methylated.

### Statistical Analysis

SPSS Version 15.0 for Windows was used for all statistical analyses. Correlations between categorical parameters were determined using Chi-square and Fisher’s exact tests.

## Results

### Clinical Characteristics of Study Patients

Between January 1, 1995 and December 31, 2005, 21 patients (42 tumors) met the criteria for this study. The clinical characteristics of these patients and tumor phenotypes are described in Table 2. All tumors were obtained by open craniotomy and partial or subtotal tumor excision. The interval between the first and second surgery ranged between one and ten years.

**Table 2:** Clinical characteristics, treatment, and outcome of patients.

Patient No.	Age at diagnosis	Sex	Pathological diagnosis	Location^a^	Treatment^b^	Age atprog.	Pathological diagnosis^c^	Treatmentafter prog.^d^	Prog.-free/survival
1	30	F	OA II	FL	OP+RT	33	OA III	T, BOP	38/91+
2	19	M	OD II	FL	OP+RT	25	OD III	–	94/129
3	39	M	OD II	FL	OP	49	OD II	RT	115/137+
4	69	F	OA II	TL	OP	71	GBM	–	30/43
5	36	M	OD III	PL	OP+RT	42	OD III	–	72/99+
6	39	M	OD II	FL	OP+RT	41	OD III	PCV	28/51
7	30	M	OD II	LV	OP+RT	34	OD II	BCNU, C, O	50/127+
8	45	F	OD II	FL	OP	47	OD II	RT	24/150
9	33	M	OA II	FL	OP+RT	37	OAII	–	43/65+
10	56	M	OA III	FL	OP	58	OAIII	RT+T	9/57+
11	63	F	OD II	FL	OP	64	OA III	–	20/27+
12	36	M	OA II	FL	OP+RT	41	OA II	–	58/109+
13	38	M	OA II	PL	OP+RT	41	AA	BCNU, O	30/71
14	37	F	OAII	FL	OP	40	OAIII	RT	31/77+
15	48	M	OAII	PL	OP+RT	50	AA	–	21/80+
16	57	M	ODIII	FL	OP	61	ODIII	RT	45/110+
17	38	M	ODII	FL	OP	40	ODII	–	27/85+
18	60	F	ODII	TL	OP	64	ODIII	RT	57/95
19	37	M	ODII	FL	OP	40	ODII	–	40/62+
20	44	F	ODIII	FL	OP	45	ODIII	T	13/25+
21	51	M	ODIII	TL	OP+RT	55	ODIII	T	47/61+

a FL = Frontal lobe, TL = Temporal lobe, LV = Lateral ventricle, PL = Parietal lobe.

b OP = operation (partial or complete resection), RT = radiotherapy.

c ODII = grade II oligodendroglioma, ODIII = grade III oligodendroglioma, OAII = grade II oligoastrocytoma, OAIII = grade III oligoastrocytoma, AA = anaplastic astrocytoma, GBM = glioblastoma multiforme.

d T = Temodal, BOP (BCNU, vincristine, cisplatin), RT = radiotherapy, PCV = (procarbazine, vincristine, CCNU), C = cisplatin, O = oncovin.

At the time of first surgery, the median age was 43.4 (19–69) years, and 14 patients (66.7%) were male. The primary tumors included nine WHO Grade II oligodendrogliomas, four anaplastic oligodendrogliomas, seven Grade II oligoastrocytomas, and one anaplastic oligoastrocytoma. For the progressive tumors, various adjuvant therapies were administered before the second surgery, including radiotherapy and chemotherapy.

Sixteen of the 21 primary tumors (76.2%) were of WHO grade II. Of the secondary tumors, only 8 (38.1%) remained WHO grade II. However, thirteen (61.9%) of the patients retained their initial histological diagnosis, and 18 (85.7%) of the tumors maintained an oligodendroglial phenotype, regardless of tumor grade. In one case (case 4), the initial grade II oligoastrocytoma progressed to a glioblastoma multiforme (GBM) tumor. In two cases (cases 13 and 15) with the initial diagnosis of grade II oligoastrocytoma, anaplastic astrocytoma was diagnosed after progression.

### Genetic Analysis by QuMA

The results from the genetic analysis of chromosomal loss are provided in Table 3. Genetic alterations were observed in 19 (90.5%) of the paired samples. At first surgery, 12 (57.1%) had 1p36 deletions, 17 (81.0%) had 19q13 deletion, and 9 (42.9%) had 1p36/19q13 codeletion. Eleven tumors (52.3%) were found to have 9p22 deletion. After progression (at second surgery) additional loss of chromosome 1p, 9p, 10q, 17p, 19q and 22q was observed in 3 (14.3%), 1 (4.8%), 3 (14.3%), 2 (9.5%), 1 (4.8%) and 3 (14.3%) cases of the 21 samples, respectively.

**Table 3:** Genetic analysis in longitudinal samples^a^.

Patient No.	Diagnosis		1p34–36				9p21–22		10q23–24				17p13		19q13				22q12				IDH1						
	1st	2nd	1st	2nd			1st	2nd	1st		2nd		1st	2nd	1st		2nd		1st		2nd		1st		2nd				
1	**OA II**	**OA III**	RET	RET			DEL	RET	RET		RET		RET	RET	DEL		RET		RET		DEL		mut		mut				
2	**OD II**	**OD III**	DEL	DEL			DEL	DEL	RET		RET		RET	DEL	DEL		DEL		RET		RET		wild		wild				
3	OD II		RET	RET			RET	RET	RET		RET		DEL	DEL	DEL		DEL		RET		DEL		wild		wild				
4	**OA II**	**GBM**	RET	RET			DEL	DEL	RET		RET		RET	RET	DEL		DEL		RET		RET		wild		wild				
5	OD III		DEL	DEL			RET	RET	RET		RET		RET	DEL	DEL		DEL		RET		RET		mut		mut				
6	**OD II**	**OD III**	DEL	DEL			DEL	DEL	RET		RET		DEL	RET	DEL		DEL		RET		RET		wild		wild				
7	OD II		DEL	DEL			DEL	DEL	RET		DEL		RET	RET	DEL		DEL		RET		RET		mut		mut				
8	OD II		RET	DEL			DEL	DEL	RET		RET		DEL	DEL	DEL		DEL		RET		RET		mut		mut				
9	OA II		DEL	RET			RET	RET	RET		RET		RET	RET	DEL		RET		RET		RET		wild		wild				
10	OA III		DEL	RET			DEL	RET	RET		RET		DEL	DEL	DEL		DEL		RET		RET		wild		wild				
11	**OD II**	**OA III**	RET	RET			DEL	DEL	DEL		RET		DEL	DEL	DEL		RET		RET		RET		mut		mut				
12	OA II		DEL	DEL			RET	RET	DEL		RET		RET	RET	RET		DEL		DEL		DEL		mut		mut				
13	**OA II**	**AA**	RET	RET			DEL	DEL	RET		DEL		DEL	DEL	RET		RET		RET		RET		mut		mut				
14	**OA II**	**OA III**	DEL	RET			RET	RET	RET		DEL		RET	RET	RET		RET		RET		DEL		mut		mut				
15	**OA II**	**AA**	RET	RET			DEL	DEL	RET		RET		DEL	RET	DEL		DEL		RET		RET		mut		mut				
16	OD III		DEL	RET			RET	RET		DEL		DEL	RET	RET		DEL		RET		RET		RET		wild		wild			
17	OD II		DEL	DEL			RET	RET		RET		RET	RET	RET		DEL		DEL		RET		RET		mut		mut			
18	**OD II**	**OD III**	DEL		DEL		RET	RET		RET		RET	DEL	RET		RET		RET		RET		RET		mut		mut			
19	OD II		RET	DEL			RET	RET		RET		RET	RET	RET		DEL		DEL		RET		RET		wild		wild			
20	OD III		DEL	DEL			RET	DEL		RET		RET	RET	RET		DEL		DEL		RET		RET		mut		mut			
21	**ODIII**	**OD III**	RET			DEL	DEL	RET		RET		RET	RET	RET		DEL		DEL		RET		RET		wild		wild			

a Genetic alterations of six tumor-related gene in paired oligodendroglial tumors. “1^st^” and “2^nd^” indicate the primary (at initial diagnosis) and secondary (after progression) resections, respectively. Diagnoses in bold indicate patients who experienced progression in tumor grade or to a more aggressive phenotype between the first and second resections. Patients with a single diagnosis are those who did not experience progression in tumor grade or phenotype, but whose diagnosis remained stable from the first to the second resection. “**DEL**”s indicate samples for which chromosomal deletion was observed, and “**RET**”s indicate samples for which retention of both chromosomal alleles was observed. “mut” = mutated IDH1; “wild” = wild type IDH1.

In some cases, a deletion which was detected in the primary tumor was not detected in the secondary tumor. For example, of the 12 cases with 1p deletion in the primary tumor, 4 (33.3%) had an intact 1p loci in the secondary tumor. Of these four tumors, three were initially oligoastrocytoma WHO grade II or III. Of the 12 cases with 1p deletion in the primary tumor, all maintained an oligodendroglial phenotype in the secondary tumor. In contrast, three of the nine cases with initially intact 1p loci were diagnosed with astrocytic tumors after progression (χ^2^ test, *P* = 0.06). Three cases had intact 1p loci in primary samples but presented with 1p deletion after progression.

Of the 11 cases with 9p deletion in primary tumors, three progressed to astrocytic tumors. Of the remaining eight cases, five of the seven with grade II primary tumors had advanced to grade III after progression. All primary tumors with intact 9p maintained their oligodendroglial phenotypes, but two of the seven grade II tumors advanced to grade III. Accordingly, 9p deletion was significantly correlated with progression to a higher grade or malignant transformation (*P*<0.05). Of the seven tumors with intact 1p but 9p deletion, three progressed to astrocytic tumors; in contrast, of the nine tumors with combined 1p and 19q loss, none progressed to astrocytic tumors (χ^2^ test, *P* = 0.03).

Of the three cases with 10q23–24 deletion in primary tumors, two (66.7%) had an intact 10q in the secondary tumors. Of the seven primary tumors with 7p13 deletion, two progressed to anaplastic astrocytomas in the subsequent tumors, and the other three progressed to grade III oligodendroglial tumors. Only one primary tumor was found with 22q12 deletion, but four secondary tumors possessed 22q12 deletions. The proportion (7 of 10) radiotherapy patients that experienced additional chromosomal loss was not significantly different from the proportion (6 of the 11) observed among patients who did not receive radiotherapy (*p*>0.05).

### IDH1 and IDH2 Mutations

**Figure 1:**
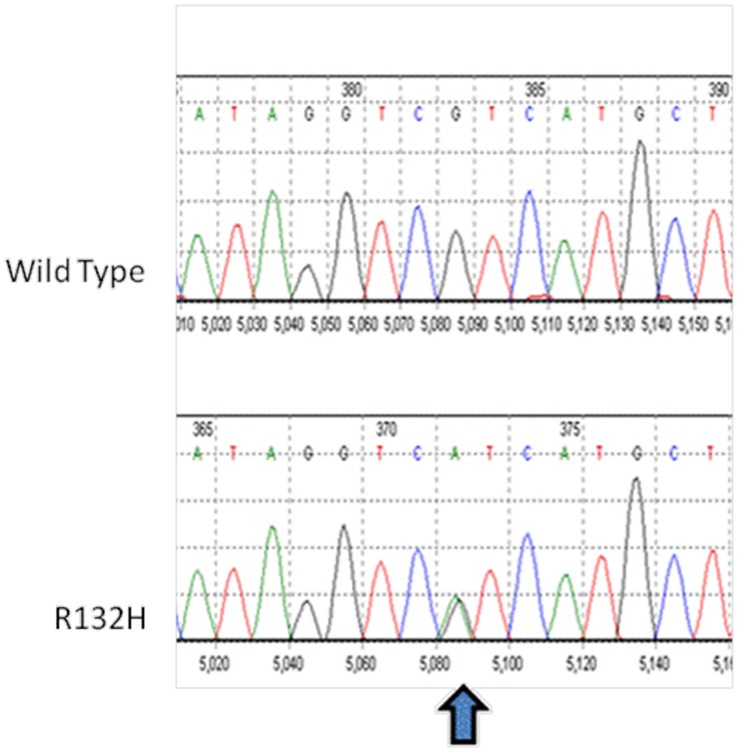
Nucleotide sequence analysis. Nucleotide sequence analysis of IDH1 showing mutation at codon 132 of IDH1 gene. Arrows indicate the position of the different nucleotide substitutions.

The results from the direct sequencing of IDH1 are provided in Table 3. At first surgery, 12 (57.1%) of tumors contained IDH1 mutations; all were heterozygous and located within the codon for amino acid residue 132. In all cases the mutation was the same, CGT→CAT, resulting in an amino acid substitution of Arg→His (Fig. 1). The IDH1 mutational status remained unchanged at second surgery in all 12 tumor pairs. No IDH2 mutations were identified in our patients with primary–progressive paired oligodendroglial tumors.

### Epigenetic Analysis

Among the primary tumors, promoter methylation of the MGMT, P16, DAPK, PTEN, RASSF1A, and Rb1 genes were detected in 38.1%, 19.0%, 38.1%, 33.3%, 66.7%, and 14.3%, respectively. At least one promoter methylation was detected in each tumor (Table 4). In secondary tumors, promoter methylation of the MGMT, P16, DAPK, PTEN, RASSF1A, and RB1 genes was detected in 52.4%, 28.6%, 38.1%, 61.9%, 66.7% and 28.6%, an addition of 3 (14.3%), 2 (9.5%), 0 (0%), 6 (28.6%), 0 (0%) and 3 (14.3%) cases, respectively.

**Table 4:** Methylation status of tumor gene promoters in longitudinal samples^a^.

Patient No.	Diagnosis		MGMT		P16		DAPK		PTEN		RASSF1A		RB1	
	1st	2nd	1st	2nd	1st	2nd	1st	2nd	1st	2nd	1st	2nd	1st	2nd
1	**OA II**	**OA III**									**+**	**+**		
2	**OD II**	**OD III**	**+**	**+**						**+**	**+**	**+**	**+**	**+**
3	OD II			**+**	**+**	**+**	**+**	**+**		**+**	**+**	**+**		
4	**OA II**	**GBM**							**+**	**+**				
5	OD III		**+**	**+**	**+**	**+**	**+**	**+**	**+**	**+**	**+**	**+**		
6	**OD II**	**OD III**	**+**	**+**			**+**	**+**	**+**	**+**	**+**	**+**		
7	OD II			**+**	**+**	**+**	**+**	**+**						
8	OD II					**+**	**+**	**+**		**+**	**+**	**+**	**+**	**+**
9	OA II						**+**	**+**		**+**		**+**		**+**
10	OA III		**+**	**+**						**+**		**+**		
11	**OD II**	**OA III**	**+**	**+**					**+**	**+**	**+**	**+**		
12	OA II			**+**	**+**	**+**			**+**	**+**	**+**	**+**		**+**
13	**OA II**	**AA**									**+**			
14	**OA II**	**OA III**	**+**	**+**					**+**	**+**				**+**
15	**OA II**	**AA**									**+**			
16	OD III						**+**	**+**			**+**	**+**		
17	OD II		**+**	**+**										
18	**OD II**	**OD III**		**+**					**+**	**+**	**+**	**+**	**+**	**+**
19	OD II						**+**	**+**		**+**	**+**	**+**		
20	OD III		**+**											
21	**OD III**	**OD III**				**+**					**+**	**+**		
Percent of samples methylated:			38.1%	52.4%	19.0%	28.6%	38.1%	38.1%	33.3%	61.9%	66.7%	66.7%	14.3%	28.6%

a Methylation status of the promoters of six tumor-related gene in paired oligodendroglial tumors. “1^st^” and “2^nd^” indicate the primary (at initial diagnosis) and secondary (after progression) resections, respectively. Diagnoses in bold indicate patients who experienced progression in tumor grade or to a more aggressive phenotype between the first and second resections. Patients with a single diagnosis are those who did not experience progression in tumor grade or phenotype, but whose diagnosis remained stable from the first to the second resection. “**+**”s indicate samples for which methylation was observed.

Of the 13 primary tumors with unmethylated MGMT promoters, 12 were grade II. Three of these progressed to astrocytic tumors, and three advanced to grade III. Of the 8 primary tumors with MGMT methylation, 6 had 1p/19q co-deletion, one had intact 1p, and another had intact 19q. Three were grade III tumors. The methylation status of MGMT was correlated with 1p deletion (*P*<0.05) and 1p/19q co-deletion (*P*<0.05), but not 19q deletion (*P* = 0.55).

Of the 14 primary tumors with unmethylated PTEN, 6 had methylated PTEN promoters after progression. Methylation of RASSF1A was most common (66.7%) among primary tumors, but two of these cases were unmethylated following progression to anaplastic astrocytomas. In one case (case 4), the tumor progressed to GBM and RASSF1A remained unmethylated. In the three patients with progression to astrocytic tumors, all tumor-related genes tested (with the exception of PTEN in case 4) were unmethylated.

Considering the effect of radiotherapy after after resection of primary tumors, 5 of the 10 patients who received radiotherapy experienced additional methylations. In contrast, 6 of the 11 patients who did not receive radiotherapy experienced additional methylations (*P*>0.05).

## Discussion

Acquisition of various genetic and epigenetic alterations may cause extensive changes in the expression of the genes involved in tumorigenesis. In this study, we have shown genetic and epigenetic changes in 21 paired oligodendroglial tumors by longitudinal assessment. The changes in some of the selected genetic loci between primary and secondary tumors have been evaluated in previous studies [2], [10]–[14], but with the exception of MGMT [11], the significance of promoter methylation in tumor-related genes remained unknown.

In this study, all the tumors experienced deletions in at least one locus, demonstrating that genetic alterations are relatively common and early events in the development of oligodendroglial tumors. Using the longitudinal samples, we observed acquisition of genetic alterations in the progressive tumors. Loss of 1p/19q is considered a common early event in the tumorigenesis of oligodendroglial tumors and has been linked to the prolonged survival times and chemosentivity of these patients [1]–[3]. However, our previous study demonstrated that only the presence of the 19q deletion, i.e., either with or without 1p deletion, was a significant predictor of longer progression-free survival in our Chinese patient population [9]. In the current study, 66.7% of primary tumors with 1p deletion retained the 1p loss after progression. In addition, two-thirds of primary tumors with 1p/19q co-deletion retained this genetic characteristic. These tumors were all oligendendrogliomas, instead of mixed tumors. This suggests that the proliferation of a sub-group of tumor cells with intact 1p or 19q may occur after the primary resection, especially in mixed oligoastrocytomas.

In the three cases with tumor progression to astrocytic tumors, their initial diagnoses were all grade II oligoastrocytoma. These comprised 37.5% of the eight grade II and III oligoastrocytomas observed among the primary tumors. Astrocytic tumors have a relatively shorter survival than oligodendroglial tumors of the same grade [21]; therefore, it is important to examine whether some characteristics such as genetic or epigenetic markers in the primary tumors can help predict progression to this phenotype. Among the astrocytic tumors, only one of the six genes examined was methylated in each of the primary tumors (PTEN in case 4; RASSF1A in cases 13 and 15), implying that epigenetic alterations in other pathways may be involved in tumorigenesis or tumor progression. All primary tumors that progressed to high-grade astrocytomas had 9p deletion, intact 1p and intact 10q; 19q deletion was detected in two of these. In addition, the two cases that progressed to anaplastic astrocytomas also displayed IDH1 mutations. Loss of chromosome 9p is less common in oligodendroglial tumors than glioblastoma multiforme tumors [9], [22], but, in glioblastomas and oligodendroglial tumors, it is associated with a shorter overall survival or progression-free survival [9], [17], [23]. Compatible with previous studies, our results also demonstrated that the tumors with combined 1p and 19q loss had a significantly lower probability of progression to astrocytic tumors. Concordantly, methylation of tumor-related gene promoters was less common in these cases, especially among secondary tumors and may suggest that these tumor-related genes are more susceptible to methylation during tumor progression or transformation.

Our results also support previous findings that 9p deletion is correlated with progression to a higher grade or malignant transformation. Located on chromosome band 9p21, p16 is a cell-cycle regulator gene. Deletion or mutation of the p16 gene is more frequently involved in high-grade gliomas than in low-grade gliomas and associated with oligodendroglioma progression from grade II to grade III [2], [24], [25]. In our cohort, we found methylation of p16 in 19% of primary and 28.6% secondary tumors. However, the methylation status of p16 was not associated with phenotype change during progression.

One of the probable targets on 10q is the tumor suppressor gene PTEN, as suggested by previous studies demonstrating that PTEN mutation was predictive of a poor outcome and shorter survival in anaplastic oligodendrogliomas [26], [27]. The gene is located at 10q23.3 and is a negative regulator of cell survival through the phosphatidylinositol 3-kinase (PI3K)/Akt pathway. PTEN methylation was detected in 6 (28.6%) secondary tumors and there was an increase from 33.3% to 61.9% observed in our study. Compared to the other genes, which remained epigenetically stable, PTEN appeared to be more susceptible to methylation during tumor progression. Loss of chromosome 10q, including the PTEN locus, is correlated with poor prognosis in patients with GBM and oligodendrogliomas [27]–[29]. Although 10q loss was less common than PTEN methylation in the secondary tumors, neither 10q nor PTEN status was correlated with changes in tumor phenotype.

In recent analyses, frequent IDH1 mutations were observed in secondary GBMs, diffuse or anaplastic astrocytomas, oligodendrogliomas, and anaplastic oligodendrogliomas [30], [31]. The IDH1 gene on chromosome 2q33 encodes the enzyme isocitrate dehydrogenase 1, which plays an important role in nicotinamide adenine dinucleotide phosphate (NADPH) production and participates in the citric acid cycle [32], [33]. When Arg is replaced, cells with decreased IDH1 expression become more susceptible to oxidative damage. These mutations appear to be associated with younger age and better outcome within each glioma entity [34], [35]. In the present study, all mutations were heterozygous and located at amino acid residue 132. The status of mutations remained unchanged in all primary–progressive paired oligodendroglial tumors.

The MGMT gene is frequently methylated in oligodendroglial tumors [9], [36], and correlation between its methylation and 1p/19q co-deletion is equivocal [36], [37]. Our results demonstrated that all three cases with progression to astrocytic tumors had an unmethylated MGMT promoter in primary tumors but subsequent MGMT methylation; MGMT methylation was correlated with isolated 1p deletion but not 19q deletion. It remains unclear whether the better survival of patients with 1p/19q co-deletion in oligodendroglial tumors can be explained by MGMT methylation.

We acknowledge the limitations of our study, which include the small number of patients, heterogeniety of treatment, and inherent bias of using methylation-specific PCR in mixed tumors. It should be noted that ten (47.6%) of our patients received radiotherapy prior to tumor progression, and it can be argued that this therapy could have affected the accumulation of genetic or epigenetic alterations. In contrast, none of our patients received chemotherapy prior to progression. Unfortunately, analysis of the relationship between radiotherapy and genetic or epigenetic alterations was limited by our relatively small sample size. Also, although the methylation-specific PCR method is a sensitive technique to identify the methylation status of genes, its application to differentiate the methylation status in single cells of tumors, especially mixed tumors, is limited. As different clonal expansion is frequently observed in progressive tumors presenting with genetic or epigenetic changes in subsequent samples, future studies should investigate changes in larger study populations and focus on the different patterns of genetic and epigenetic alterations in tumors with intra-tumoral heterogeneity.

Using a longitudinal assessment, this study identified genetic and epigenetic alterations in 21 primary-progressive paired oligodendroglial tumors. In the primary tumors that experienced subsequent progression to high-grade astrocytomas, all had 9p deletion, intact 1p, intact 10q and unmethylated MGMT. These results require confirmation in a larger study population, but they may indicate a molecular signature for identifying tumors at risk of progression to the astrocytic phenotype. Patients with this signature should be monitored closely. Also, the safety, efficacy and timing of the use of chemotherapy or radiotherapy should be investigated in these patients.

## References

[1] Smith JS, Perry A, Borell TJ, Lee HK, O'Fallon J, et al (2000) Alterations of chromosome arms 1p and 19q as predictors of survival in oligodendrogliomas, astrocytomas, and mixed oligoastrocytomas. J Clin Oncol 18: 636–645. 10653879 10.1200/JCO.2000.18.3.636

[2] Fallon KB, Palmer CA, Roth KA, Nabors LB, Wang W, et al (2004) Prognostic value of 1p, 19q, 9p, 10q, and EGFR-FISH analyses in recurrent oligodendrogliomas. J Neuropathol Exp Neurol 63: 314–322. 15099021 10.1093/jnen/63.4.314

[3] Dehais C, Laigle-Donadey F, Marie Y, Kujas M, Lejeune J, et al (2006) Prognostic stratification of patients with anaplastic gliomas according to genetic profile. Cancer 107: 1891–1897. 16986124 10.1002/cncr.22211

[4] Yan H, Parsons DW, Jin G, McLendon R, Rasheed BA, et al (2009) IDH1 and IDH2 mutations in gliomas. N Engl J Med 360: 765–773. 19228619 10.1056/NEJMoa0808710 PMC2820383

[5] Reitman ZJ, Yan H (2010) Isocitrate dehydrogenase 1 and 2 mutations in cancer: alterations at a crossroads of cellular metabolism. J Natl Cancer Inst 102: 932–941. 20513808 10.1093/jnci/djq187 PMC2897878

[6] Pegg AE (1990) Mammalian O6-alkylguanine-DNA alkyltransferase: regulation and importance in response to alkylating carcinogenic and therapeutic agents. Cancer Res 50: 6119–6129. 2205376

[7] Alonso ME, Bello MJ, Gonzalez-Gomez P, Arjona D, Lomas J, et al (2003) Aberrant promoter methylation of multiple genes in oligodendrogliomas and ependymomas. Cancer Genet Cytogenet 144: 134–142. 12850376 10.1016/s0165-4608(02)00928-7

[8] Hegi ME, Diserens AC, Gorlia T, Hamou MF, de Tribolet N, et al (2005) MGMT gene silencing and benefit from temozolomide in glioblastoma. N Engl J Med 352: 997–1003. 15758010 10.1056/NEJMoa043331

[9] Kuo LT, Kuo KT, Lee MJ, Wei CC, Scaravilli F, et al (2009) Correlation among pathology, genetic and epigenetic profiles, and clinical outcome in oligodendroglial tumors. Int J Cancer 124: 2872–2879. 19330828 10.1002/ijc.24303

[10] Walker C, Haylock B, Husband D, Joyce KA, Fildes D, et al (2006) Clinical use of genotype to predict chemosensitivity in oligodendroglial tumors. Neurology 66: 1661–1667. 16769937 10.1212/01.wnl.0000218270.12495.9a

[11] Lavon I, Zrihan D, Zelikovitch B, Fellig Y, Fuchs D, et al (2007) Longitudinal assessment of genetic and epigenetic markers in oligodendrogliomas. Clin Cancer Res 13: 1429–1437. 17332285 10.1158/1078-0432.CCR-06-2050

[12] Campbell BA, Horsman DE, Maguire J, Young S, Curman D, et al (2008) Chromosomal alterations in oligodendroglial tumours over multiple surgeries: is tumour progression associated with change in 1p/19q status? J Neurooncol 89: 37–45. 18458822 10.1007/s11060-008-9597-2

[13] Jeuken JW, Sijben A, Bleeker FE, Boots-Sprenger SH, Rijntjes J, et al (2011) The nature and timing of specific copy number changes in the course of molecular progression in diffuse gliomas: further elucidation of their genetic “life story”. Brain Pathol 21: 308–320. 21029244 10.1111/j.1750-3639.2010.00447.x PMC8094293

[14] Lass U, Nümann A, von Eckardstein K, Kiwit J, Stockhammer F, et al (2012) Clonal analysis in recurrent astrocytic, oligoastrocytic and oligodendroglial tumors implicates IDH1- mutation as common tumor initiating event. PLoS One 7: e41298. 22844452 10.1371/journal.pone.0041298 PMC3402513

[15] Nigro JM, Takahashi MA, Ginzinger DG, Law M, Passe S, et al (2001) Detection of 1p and 19q loss in oligodendroglioma by quantitative microsatellite analysis, a real-time quantitative polymerase chain reaction assay. Am J Pathol 158: 1253–1262. 11290543 10.1016/S0002-9440(10)64076-X PMC1891922

[16] Wolter M, Reifenberger J, Blaschke B, Ichimura K, Schmidt EE, et al (2001) Oligodendroglial tumors frequently demonstrate hypermethylation of the CDKN2A (MTS1, p16INK4a), p14ARF, and CDKN2B (MTS2, p15INK4b) tumor suppressor genes. J Neuropathol Exp Neurol 60: 1170–1180. 11764089 10.1093/jnen/60.12.1170

[17] Schmidt MC, Antweiler S, Urban N, Mueller W, Kuklik A, et al (2002) Impact of genotype and morphology on the prognosis of glioblastoma. J Neuropathol Exp Neurol 61: 321–328. 11939587 10.1093/jnen/61.4.321

[18] Schmidt EE, Ichimura K, Reifenberger G, Collins VP (1994) CDKN2 (p16/MTS1) gene deletion or CDK4 amplification occurs in the majority of glioblastomas. Cancer Res 54: 6321–6324. 7987821

[19] Herman JG, Graff JR, Myohanen S, Nelkin BD, Baylin SB (1996) Methylation-specific PCR: a novel PCR assay for methylation status of CpG islands. Proc Natl Acad Sci USA 93: 9821–9826. 8790415 10.1073/pnas.93.18.9821 PMC38513

[20] Ichimura K, Schmidt EE, Goike HM, Collins VP (1996) Human glioblastomas with no alterations of the CDKN2A (p16INK4A, MTS1) and CDK4 genes have frequent mutations of the retinoblastoma gene. Oncogene 13: 1065–1072. 8806696

[21] Ohgaki H, Kleihues P (2005) Population-based studies on incidence, survival rates, and genetic alterations in astrocytic and oligodendroglial gliomas. J Neuropathol Exp Neurol 64: 479–489. 15977639 10.1093/jnen/64.6.479

[22] Brat DJ, Seiferheld WF, Perry A, Hammond EH, Murray KJ, et al (2004) Analysis of 1p, 19q, 9p, and 10q as prognostic markers for high-grade astrocytomas using fluorescence in situ hybridization on tissue microarrays from Radiation Therapy Oncology Group trials. Neuro Oncol 6: 96–103. 15134623 10.1215/S1152851703000231 PMC1871985

[23] Cairncross JG, Ueki K, Zlatescu MC, Lisle DK, Finkelstein DM, et al (1998) Specific genetic predictors of chemotherapeutic response and survival in patients with anaplastic oligodendrogliomas. J Natl Cancer Inst 90: 1473–1479. 9776413 10.1093/jnci/90.19.1473

[24] Barker FG, Chen P, Furman F, Aldape KD, Edwards MS, et al (1997) P16 deletion and mutation analysis in human brain tumors. J Neurooncol 31: 17–23. 9049826 10.1023/a:1005768910871

[25] Bigner SH, Matthews MR, Rasheed BK, Wiltshire RN, Friedman HS, et al (1999) Molecular genetic aspects of oligodendrogliomas including analysis by comparative genomic hybridization. Am J Pathol 155: 375–386. 10433931 10.1016/S0002-9440(10)65134-6 PMC1866844

[26] Ino Y, Betensky RA, Zlatescu MC, Sasaki H, Macdonald DR, et al (2001) Molecular subtypes of anaplastic oligodendroglioma: implications for patient management at diagnosis. Clin Cancer Res 7: 839–845. 11309331

[27] Sasaki H, Zlatescu MC, Betensky RA, Ino Y, Cairncross JG, et al (2001) PTEN is a target of chromosome 10q loss in anaplastic oligodendrogliomas and PTEN alterations are associated with poor prognosis. Am J Pathol 159: 359–367. 11438483 10.1016/S0002-9440(10)61702-6 PMC1850425

[28] Burton EC, Lamborn KR, Feuerstein BG, Prados M, Scott J, et al (2002) Genetic aberrations defined by comparative genomic hybridization distinguish long-term from typical survivors of glioblastoma. Cancer Res 62: 6205–6210. 12414648

[29] Trost D, Ehrler M, Fimmers R, Felsberg J, Sabel MC, et al (2007) Identification of genomic aberrations associated with shorter overall survival in patients with oligodendroglial tumors. Int J Cancer 120: 2368–2376. 17285580 10.1002/ijc.22574

[30] Hartmann C, Meyer J, Balss J, Capper D, Mueller W, et al (2009) Type and frequency of IDH1 and IDH2 mutations are related to astrocytic and oligodendroglial differentiation and age: a study of 1,010 diffuse gliomas. Acta Neuropathol 118: 469–474. 19554337 10.1007/s00401-009-0561-9

[31] Kloosterhof NK, Bralten LB, Dubbink HJ, French PJ, van den Bent MJ (2010) Isocitrate dehydrogenase-1 mutations: a fundamentally new understanding of diffuse glioma? Lancet Oncol 12: 83–91. 20615753 10.1016/S1470-2045(10)70053-X

[32] Narahara K, Kimura S, Kikkawa K, Takahashi Y, Wakita Y, et al (1985) Probable assignment of soluble isocitrate dehydrogenase (IDH1) to 2q33.3. Hum Genet 71: 37–40. 3861566 10.1007/BF00295665

[33] Hartong DT, Dange M, McGee TL, Berson EL, Dryja TP, et al (2008) Insights from retinitis pigmentosa into the roles of isocitrate dehydrogenases in the Krebs cycle. Nat Genet 40: 1230–1234. 18806796 10.1038/ng.223 PMC2596605

[34] Houillier C, Wang X, Kaloshi G, Mokhtari K, Guillevin R, Laffaire J, et al (2010) IDH1 or IDH2 mutations predict longer survival and response to temozolomide in low-grade gliomas. Neurology 75: 1560–1566. 20975057 10.1212/WNL.0b013e3181f96282

[35] Sanson M, Marie Y, Paris S, Idbaih A, Laffaire J, Ducray F, et al (2009) Isocitrate dehydrogenase 1 codon 132 mutation is an important prognostic biomarker in gliomas. J Clin Oncol 27: 4150–4154. 19636000 10.1200/JCO.2009.21.9832

[36] Jha P, Suri V, Jain A, Sharma MC, Pathak P, et al (2010) O6-methylguanine DNA methyltransferase gene promoter methylation status in gliomas and its correlation with other molecular alterations: first Indian report with review of challenges for use in customized treatment. Neurosurgery 67: 1681–1691. 21107199 10.1227/NEU.0b013e3181f743f5

[37] Ferrer-Luna R, Nunez L, Piquer J, Arias E, Dasi F, et al (2011) Whole-genomic survey of oligodendroglial tumors: correlation between allelic imbalances and gene expression profiles. J Neurooncol 103: 71–85. 20820872 10.1007/s11060-010-0369-4

